# Rehabilitation Professional and Patient Satisfaction with Telerehabilitation of Musculoskeletal Disorders: A Systematic Review

**DOI:** 10.1155/2022/7366063

**Published:** 2022-08-02

**Authors:** Junaid Amin, Basaruddin Ahmad, Salman Amin, Ammar Ahmed Siddiqui, Mohammad Khursheed Alam

**Affiliations:** ^1^School of Dental Sciences, Health Campus, University Sains Malaysia, Kubang Kerian, Malaysia; ^2^College of Medicine & Dentistry, University of Lahore, Lahore, Pakistan; ^3^College of Dentistry, Preventive Dental Sciences, University of Hail, Saudi Arabia; ^4^Department of Community Dentistry, College of Dentistry, Bakhtawar Amin Medical and Dental College, Multan, Pakistan; ^5^Department of Preventive Dentistry, College of Dentistry, Jouf University, Sakaka, Aljouf, Saudi Arabia

## Abstract

Telerehabilitation offers an alternative healthcare delivery remotely in a patient's environment at a lower cost, better accessibility, and equivalent quality to the standard approach. Several studies had examined the effectiveness of telerehabilitation inpatients with musculoskeletal disorders, and although there is evidence that it is at least equally effective as the standard care, the patient and rehabilitation professional satisfaction with the delivery method is not conclusive. A systematic review was conducted to study the patients' and rehabilitation professionals' satisfaction with telerehabilitation for musculoskeletal disorders. A search for relevant studies on 29 April 2021 was carried out in Medline/PubMed, Scopus, and Web of Science (WOS). The search terms included “telerehabilitation,” AND “satisfaction” AND “musculoskeletal disorders,” “telehealth,” “telemedicine,” “patient experience,” and “pain”. Fifteen eligible studies with 12,341 patients were included in this systematic review. A report was included if it (a) assessed the satisfaction of patients or professionals or both as one of the outcomes of a telerehabilitation intervention, (b) included adults 18 years and above with musculoskeletal disorders, and (c) is an intervention study using a quantitative approach. The quality of studies was assessed using the critical appraisal checklist tool developed by Joanna Briggs Institute (JBI). Most of the studies reported that patients were satisfied with both telerehabilitation and face-to-face intervention. However, few studies reported that patients were more satisfied with telerehabilitation compared to face-to-face of intervention. Patients in one study had preferred the incorporation of telerehabilitation and face-to-face sessions. Two of three studies had reported overall satisfaction with telerehabilitation by the professionals. Overall, there is evidence that patients and rehabilitation professional are satisfied with telerehabilitation compared to face-to-face consultation.

## 1. Introduction

Telerehabilitation is the provision of rehabilitation services from a provider to a patient via a telecommunication system and information technology [1]. Such an alternative approach attracts the attention of the healthcare community because of several advantages. It saves time and cost related to travelling to and waiting at the healthcare centre, facilitates and improves access to services, and promises equal quality services to the public [2]. Telerehabilitation has been shown to be successfully performed and effective for people with medical conditions such as stroke, breast cancer patients, cardiopulmonary, and musculoskeletal disorders (MSDs) [3–6].

Patient satisfaction is one of the secondary but very important outcomes in healthcare delivery. Patients are the main source of information to report on the quality of service provided, that is whether the standards of patient care and treatment received met the expectations. Telehealth platform changes with advancing technology and the mode of delivery can change dramatically; for example, from voice to video to multiperson conference, but regardless of the changes, a consistent patient-provider relationship must be formed [7]. As telehealth use increases, it becomes increasingly important to maintain patient satisfaction similar to the standard approach. Telehealth developers should be flexible to accommodate the patients' and healthcare professionals' perspective and needs; these should be monitored regularly. The success of a telehealth program heavily relies on patient satisfaction [7]. A variety of components underlying the satisfaction have been addressed in different studies that may include overall satisfaction and satisfaction with the application and services [8–10]. Moreover, to achieve optimal outcomes of the treatment and satisfaction of the patient, it is imperative to maintain a strong relationship between patient and professionals [11]. Therefore, it is important to take the satisfaction of both patients and the professionals for a successful and sustainable telehealth programs.

A recent systematic review reported that the evidence for the efficacy of telehealth interventions in improving musculoskeletal pain-related outcomes is comparable to the standard face-to-face interventions [12]. According to healthcare workers, online services can be a helpful addition to face-to-face therapies for chronic pain. Patients are also enthusiastic about telehealth approaches to healthcare delivery [7]. Patients who received exercises through telerehabilitation after shoulder joint replacement reported feelings of “closeness at a distance,” freedom, and increased awareness about their “body and self” [13]. Likewise, there were good levels of patient satisfaction with telehealth delivery for cognitive behavioral therapy, exercise, and pain-coping interventions [14].

A broad range of research on telemedicine, or immersive video consultations, has been conducted in various locations around the world. Telemedicine commentators often emphasize the need for more research on the protection, efficacy, and cost-effectiveness of healthcare delivery. As a result of the abundance of publications about patient satisfaction in the telemedicine literature, which are overwhelmingly optimistic, there is a tendency to believe that research in telerehabilitation is relatively of a less priority and a new area that should be focused. There is a good body of literature that examined the patient and professional satisfaction with telerehabilitation. It is imperative to take the perspectives of patients to increase the access, acceptance, and adherence with telerehabilitation in MSD management. To date, there has been no comprehensive systematic review on the satisfaction of patients and professionals with telerehabilitation for musculoskeletal disorder management. The current systematic review is aimed at determining the satisfaction of patients and professionals with telerehabilitation as compared to traditional face-to-face intervention for musculoskeletal disorders.

## 2. Methods

We follow the PRISMA guidelines to conduct this systematic review [15]. The PRISMA 2020 checklist can found in supplementary file 1. The study protocol was registered with PROSPERO (CRD42021252078).

### 2.1. Review Question

Based on PRISMA guidelines, the search questions were built on PICO format as follows: are professionals and patients satisfied with the telerehabilitation as compared to the face-to-face rehabilitation of musculoskeletal disorders? PICO format for the review questions is explained as follows: the population (patients with musculoskeletal issues)/(professionals dealing musculoskeletal issues), the intervention (telerehabilitation), comparison (face-to-face), and outcome (satisfaction).

### 2.2. Search Strategy

We searched the electronic databases from January 1980 to April 2021 reports in the English language. Databases searched include PubMed, Scopus, and Web of Science (WOS). The search terms were “telerehabilitation”, “satisfaction,” and “musculoskeletal disorders.” We also included common synonyms: “telehealth,” “telemedicine,” “patient experience,” and “expectations,” “pain.” Additional searches were carried out on Google Scholar and ResearchGate platforms. The search string for each variable has been provided as supplementary file 2. Moreover, the references of the included studies were also explored to find the relevant literature meeting the inclusion criteria. Two authors (JA and AAS) independently screened the abstracts based on the inclusion and exclusion criteria.

### 2.3. Inclusion and Exclusion Criteria

A report was included if it (a) assessed the satisfaction of patients or professionals or both as one of the outcomes of a telerehabilitation intervention, (b) included adults 18 years and above with musculoskeletal disorders, and (c) is an intervention study using a quantitative approach. A report was excluded if it is (a) not in English language and if they are (b) review articles, case reports, qualitative studies, book chapters, and articles with low quality.

## 3. Results

### 3.1. Study Selection

**Figure 1:**
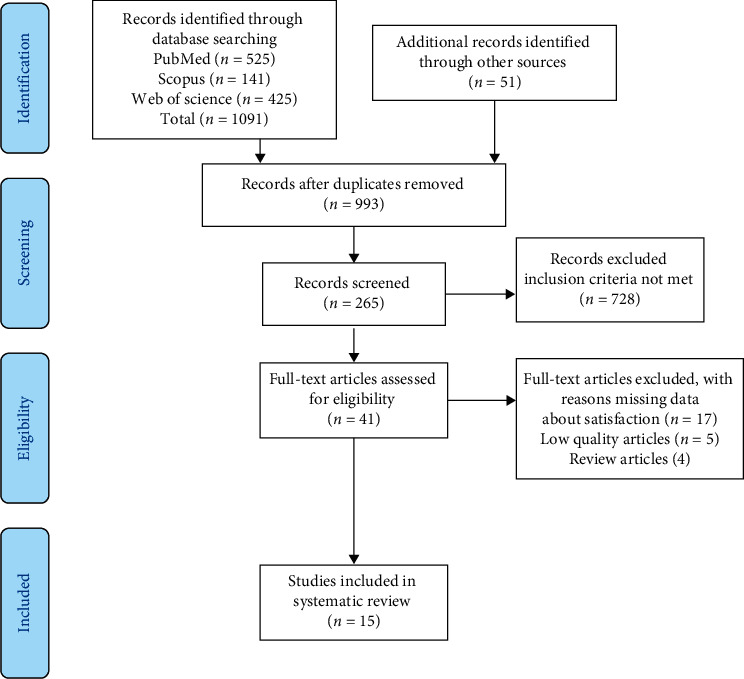
PRISMA flow diagram showing study identification and selection process.

A total of 1091 studies were identified from Scopus (*n* = 141), Web of Science (*n* = 425), and PubMed (*n* = 525). An additional 51 reports were identified from Google Scholar, Research Gate, and reference list of the searched articles. A total of 149 duplicate studies were removed using the Reference Management Software Package (Endnote X9). A total of 728 were excluded because the studies did not meet the inclusion (satisfaction was not assessed = 291, not within the age range = 15, and not intervention study = 314) and exclusion (non-English report = 5 and not relevant report = 103) criteria. The full text of 41 studies was reviewed, and 26 studies were excluded due to missing data about satisfaction (*n* = 17), low-quality articles (*n* = 5), and review articles (*n* = 4). A total of 15 studies were included in systematic review after ensuring that those are fulfilling the inclusion criteria of the study.  Figure 1 shows a schematic of the study identification and selection process.

### 3.2. Data Abstraction

Two field-based experts (JA and AAS) reviewed the full text of the report independently and used a data extraction form to record information relating to the author, year of the report, location of study, number of subjects, study design, methodology, study population, technology explored, and measures of satisfaction and findings of the study. Cohen's kappa for inter rater reliability was calculated as.74 showing substantial agreement. Disagreements were resolved by consensus and discussion or involved arbitration by the last author (MKA).

### 3.3. Synthesis of Results

In our review, content analysis was performed to synthesize the results. We examined articles that reported patient and professionals satisfaction. The different parameters of satisfaction were categorized into overall satisfaction, satisfaction with application, satisfaction with services, satisfaction, and patient-provider relationship. We performed a physical count of these variables to find the consistency. All parameters were displayed in number of occurrence. Moreover, the heterogeneity was also observed including sample size, study design, risk of bias, setting of studies, and outcome measures.

### 3.4. Quality Assessment

**Figure 2:**
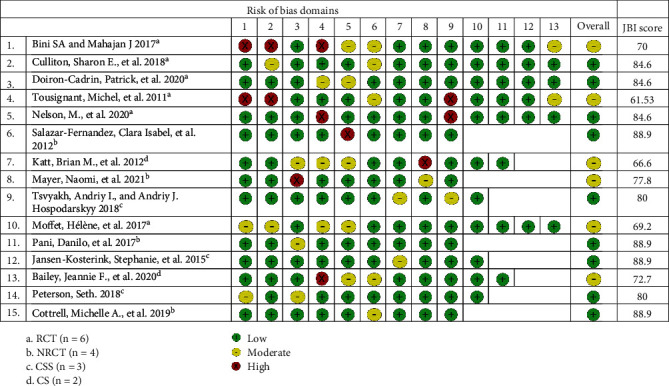
Risk of bias assessment based on Joanna Briggs Institute (JBI) critical appraisal tool.

We utilized tools developed by the Joanna Briggs Institute (JBI) critical appraisal checklist to assess the quality of RCTs, case-control studies, cohort studies, non-RCTs, and case series [16, 17]. The JBI has well-established reliability and validity to assess the risk of bias in studies [16, 18]. Two authors independently evaluated the quality of each study. The JBI score was calculated for each study using the checklist, and score was presented as percentages. The study with a JBI score of 20-49% was considered high risk of bias and with 50-79% and 80-100% moderate and low risk of bias, respectively (Figure 2).

### 3.5. Article Characteristics

There were 15 eligible studies with a total of 12,341 participants. Sample sizes ranged from 3 to 10264 patients. All studies were from the developed countries including five from the United States, four from Europe, three from Canada, two from Australia, and one from Israel. There were 6 RCTs, 3 non-RCTs, 3 case-series studies, and 2 cohort studies and 1 quasiexperimental trial. Most studies (*n* = 10) used asynchronous mode through while others used synchronous videoconference mode (*n* = 4) and a combination of both (*n* = 1).

### 3.6. Patient Satisfaction

The majority of the studies included satisfaction of telerehabilitation as one of the secondary objectives (*n* = 11), and few studies included satisfaction in their primary objective (*n* = 4). All the included studies reported the findings on patient satisfaction. Patient satisfaction was reported in both the intervention (telerehabilitation) and face-to-face groups.

The measures of satisfaction include overall satisfaction, satisfaction with application, satisfaction with services, and patient-provider relationship. A broad range of satisfaction was assessed and ranged from overall satisfaction (*n* = 6), satisfaction with application (*n* = 4), satisfaction with services (*n* = 4), satisfaction and patient-provider relationship (*n* = 11). These parameters were assessed in percentages.

The majority of the studies reported that patients were satisfied with both telerehabilitation and face-to-face [8–10, 19–22]. Three studies reported that patient satisfaction with telerehabilitation was significantly higher than that with face-to-face care [13, 21, 23]. One study reported that the patients preferred having an incorporated program that includes both telerehabilitation and face-to-face sessions [24] (Table 1).

### 3.7. Professional Satisfaction

There was evidence from the studies for the satisfaction of professionals with telerehabilitation. Of 15 included studies, only 3 (20%) studies assessed the satisfaction of the professionals, 2 of those had reported that there was satisfaction of professionals with telerehabilitation services [8, 22]. However, a study reported less satisfaction when dealing with new patients and favored a blended program including face-to-face visits and telerehabilitation [24] (Table 2).

### 3.8. Risk of Bias

Based on Joanna Briggs Institute (JBI) critical appraisal tool, the majority of the studies (*n* = 9) were categorized as having a low risk of bias and 6 studies, moderate risk of bias. Details about the risk of bias are shown in Figure 2.

## 4. Discussion

The current systematic review found that patients in the majority of the studies are satisfied with both telerehabilitation and conventional delivery method of treatment for MSD [8–10, 19–22]. Satisfaction with telehealth approach also has been reported in systematic reviews on teledermatology [30], telepsychiatry [31], and telemedicine for stroke [32].

The current review is providing promising results about satisfaction that are in line with the other clinical cases dealt with telerehabilitation. In poststroke telerehabilitation, professionals and patients reported high level of satisfaction and acceptance with remote intervention through Internet-based video conference in their home setting [3, 33]. Moreover, a study on stroke survivors reported a better relationship of patients with their providers in telegroup [34]. Patient satisfaction with telerehabilitation is imperative as it influences compliance and motivation towards intervention as patients are more engaged with their rehab professionals and confidence level is enhanced.

The high level of satisfaction with telerehabilitation among the professionals found in two studies is consistent with earlier reports [30, 32]. Three areas of satisfaction assessed, patient performance, patient-provider relationship, and quality of technology are related to the capability of professionals to adapt and accept of the new technology in their practice. However, professionals in one study are less satisfied with telerehabilitation when dealing with new patients but were satisfied with it in the follow-up sessions as they reported that certain conditions are hard to asses through telehealth when a radiograph is needed [24].

The above findings suggest that telehealth is an accepted service delivery method by patients and health care professionals but it is not without limitation. It may not be suitable for an initial consultation because in some cases physical examination, blood sample, and radiographic imaging are required to make a definitive diagnosis but more suitable for follow-up sessions. Telerehabilitation is more effective if a patient's record is available to the provider during a consultation session. A combination of face-to-face and telehealth consultation is perhaps a reasonable option to address the issue.

Apart from the advantages of telerehabilitation in reducing cost, time, and disparities in access and satisfying the expectation of patients and professionals [35], it also serves as a rational option for service delivery method during the current COVID-19 pandemic. The health authorities are opting the telehealth to prevent the spread of infection among communities [36, 37]. The approach reduces the number of physical visit and contact between patients and other individuals during travel and at the healthcare centre and thus lowers the risk of infection [38]. As the health professional and patient relationship is a core element for successful treatment using telehealth, the gap has to be reduced by improving the communication skill of the provider and instilling the confidence of patients in the professionals [39]. Other issues that need consideration in improving the relationship include feasibility, acceptability, and utility [40]. These core factors indicate the willingness and capabilities of professionals to deliver the healthcare services to their patients. Accessibility, awareness, and skills relating the teletechnology among patients also influence the usage and success of telehealth delivery. The patient's expectations are also met with telerehabilitation [41]. Hence, it is imperative to enhance the efforts to use e-health technology in rehabilitation settings.

This is the first systematic review relating to satisfaction with telerehabilitation that is providing sound scientific knowledge to the body of literature and useful for policy-makers for future clinical decision-making. However, it has some limitations; thus, interpretation of the findings should be made with caution. There is wide variation in the measures of assessment and reporting of patients and professionals' satisfaction despite the evidence being supportive of telerehabilitation. In this systematic review, we found heterogeneity in terms of study design, setting of studies, and outcome measures but there is consistency in the satisfaction and effectiveness with telerehabilitation services Meta-analysis was not performed due to pooling of the results of different outcome measures which could lead to heterogeneity and bias meta-analysis [42]. The evidence thus far is from the developed countries, and its performance in the developing and underdeveloped countries has yet been reported; thus, further research on telerehabilitation in these countries is recommended.

## 5. Conclusion

In our systematic review, we contributed a substantial amount of literature about satisfaction with telerehabilitation that is relatively a new area to be explored. The findings of this review point out that telerehabilitation has similar level of satisfaction that is comparable with a face-to-face consultation among patient and professionals alike.

## Figures and Tables

**Table 1:** Patient satisfaction with telerehabilitation.

	Authors (years)	Country	Study design & quality of evidence^a^	Sample size	Study papulation	Technology explored	Variables assessed	Study demonstrate satisfaction	Findings
1	Bini and Mahajan, 2017 [10]	USA	RCT,1	28 (intervention, *n* = 13; face-to-face, *n* = 15)	Post TKA	Asynchronous video-based mobile application	(i) Satisfaction with the experience (ii) Overall satisfaction	Yes (TR = FTF)	Patient satisfaction was high for both groups with no difference
2	Culliton et al., 2018 [19]	Canada	RCT,1	319 (intervention, *n* = 154; face-to-face, *n* = 165)	Post TKA	Online e-learning tool	(i) Patient Acceptable Symptom State (PASS) question (ii) Satisfaction with current health state	Yes (TR = FTF)	78.6% in the intervention and 78.2% in the control groups were satisfied
3	Doiron-Cadrin et al., 2020 [20]	Canada	RCT,1	22 (intervention, *n* = 11; face-to-face, *n* = 11)	Pre TKA/THA	Reacts lite application	Telecommunication applications	Yes (TR = FTF)	Patient satisfaction was excellent toward telerehabilitation without any significant association between the groups
4	Tousignant et al., 2011 [8]	Canada	RCT,1	42 (intervention, *n* = 22; face-to-face, *n* = 20)	Post TKA	Video conferencing	(i) Healthcare satisfaction questionnaire (ii) Satisfaction with the relationship with the healthcare professional (iii) Satisfaction with the services delivered, (iv) Satisfaction with the general healthcare organization	Yes (TR = FTF)	Patient satisfaction was observed in both groups (tele and comparison) without any significant association between the groups
5	Nelson et al., 2020 [21]	Australia	RCT,1	69 (intervention, *n* = 34; face-to-face, *n* = 35)	THA	eHAB application	(i) Satisfaction questionnaire of the rehabilitation program (ii) Overall satisfaction	Yes (TR > FTF)	In both groups (intervention & tele), overall satisfaction was high but the score regarding ease of attending appointment was high in the intervention group as compared to telegroup
6	Salazar-Fernandez et al., 2012 [25]	Spain	NRCT,2	992 (intervention, *n* = 282; face-to-face, *n* = 710)	Temporomandibular joint disorders	Store-and-forward telemedicine system (SFTMS)	(i) Telemedicine again (ii) Feel uncomfortable (iii) Prefer to be treated quickly (iv) Travel saving (v) Hours of lost-work saving	Yes (TR = FTF)	283 patients reported that they would like to consult by teleconsultation again There was no record of uncomfortable incidence from any patient with teleconsultation
7	Katt et al., 2012 [24]	USA	PCS,2	167 (follow-up, *n* = 111; new, *n* = 56)	Upper extremity conditions	A telephone call or video	(i) Quality of time spent with the provider, preference of telehealth versus an in office encounter, or a combination of both	Yes (TR = FTF)	Both patient and physician responded that they are very satisfied with the telehealth sessions but some patients should have telerehabilitation program incorporated with some face-to-face evaluation sessions as they were less satisfied while evaluating new patients
8	Mayer et al., 2021 [26]	Israel	NRCT,2	18 (intervention, *n* = 9; face-to-face, *n* = 9)	Upper limb function after fractures	Biofeedback system of elbow motion	(i) Patient satisfaction questionnaire	Yes (TR = FTF)	The telerehabilitation group reported a higher level of enjoyment of the self-practice with less support from family members and no difference was found regarding satisfaction level between two groups
9	Tsvyakh, 2018 [23]	Ukraine	CSS,4	74 (intervention, *n* = 48; face-to-face, *n* = 26)	Injuries of the lower extremities	Home remote monitoring by using a smartphone	(i) Overall satisfaction	Yes (TR > FTF)	Patient satisfaction was higher for the telerehabilitation as compared to traditional rehabilitation due to the reason of time saving and cost of rehabilitation
10	Moffet et al., 2017 [9]	Canada	RCT,1	182 (intervention, *n* = 84; face-to-face, *n* = 98)	Post TKA	Videoconferencing system	(i) Relationship with the professional (ii) Delivery of services (iii) Organization of services	Yes (TR = FTF)	Overall satisfaction was high in both groups without any difference in both groups
11	Pani et al., 2017 [13]	Italy	NRCT,2	40 (intervention, *n* = 20; face-to-face, *n* = 20)	Hand function impairment in rheumatic patients	Sensorized tools for hand exercises	(i) Satisfaction of the product (ii) Associated services	Yes (TR > FTF)	Most of the patients were satisfied with the services and accepted the telerehabilitation system
12	Jansen-Kosterink et al., 2015 [27]	Netherlands	CSS,4	60 (intervention, *n* = 41; face-to-face, *n* = 19)	CLBP	Teleconference	(i) Rate satisfaction with service (ii) Recommend the service to another patient	Yes (TR = FTF)	About 70% telerehabilitation scored 6 or higher score on a scale from 0 to 10 and 36% reported that they would like to recommend the telerehabilitation services to another patient
13	Bailey et al., 2020 [28]	USA	RCS,3	Back pain (*n* = 6468), knee pain (*n* = 3796)	Low back & knee pain	Hinge health app installed	Overall satisfaction	Yes (TR = FTF)	There was an overall satisfaction among patients in Digital Care Program (DCP) with a final satisfaction score of 8.97/10
14	Peterson, 2018 [29]	USA	CSS,4	3	CLBP	Mobile phone application with synchronous audio and video booster sessions	Overall satisfaction	Yes (TR = FTF)	The overall satisfaction with the telerehabilitation program was very high
15	Cottrell et al., 2019 [22]	Australia	NRCT,2	61 (intervention, *n* = 46; face-to-face, *n* = 15)	LBP & neck pain	Mobile phone application	(i) Cost (ii) Access (iii) Time (iv) Overall satisfaction	Yes (TR = FTF)	(i) The overall satisfaction between two groups was similar (ii) The satisfaction level was high for responses relating to access, cost and time saving, and overall experience in TR group (iii) The satisfaction of professionals regarding appointments was averaged 4.1 out of 5 points

TR: telerehabilitation; FTF: face-to-face; RCT: randomized clinical trial; NRCT: nonrandomized clinical trial; CCS: case-control study; CSS: case-series study; RCS: retrospective cohort study; PCS: prospective cohort study CLBP: chronic low back pain; LBP: low back pain; TKA: total knee arthroplasty; THA: total hip arthroplasty. ^a^Level of evidence. (1) Properly designed RCT or systematic review with meta-analysis; (2) well-designed controlled trial without randomization, prospective study, or comparative cohort trial; (3) case-control study or retrospective cohort study; and (4) case-series or cross-sectional study.

**Table 2:** Professional satisfaction with telerehabilitation.

	Authors (years)	Country	Quality of evidence^a^	Sample size	Study papulation	Technology explored	Type of provider	Overall satisfaction	Findings
1	Tousignant et al., 2011 [8]	Canada	RCT,1	42 (intervention, *n* = 22; face-to-face, *n* = 20)	Post-TKA	Video conferencing	Physiotherapists	Yes	Physiotherapists were highly satisfied regarding the following: (i) Goal achievement (ii) Patient-therapist relationship (iii) Overall session satisfaction (iv) Quality and performance of the technological platform
2	Katt et al., 2012 [24]	USA	RCS,3	167 (follow-up, *n* = 111; new, *n* = 56)	Upper extremity conditions	A telephone call or video	Physicians	Yes	Physician responded that they are very satisfied with the telehealth sessions but reported that some patients should have telerehabilitation program incorporated with some face-to-face evaluation sessions, as they were less satisfied while evaluating new patients
3	Cottrell et al., 2019 [22]	Australia	NRCT,2	61 (intervention, *n* = 46; face-to-face, *n* = 15)	LBP & neck pain	Mobile phone application	Rehab professionals	Yes	The satisfaction of professionals regarding appointments was high (averaged 4.1 out of 5 points)

TR: telerehabilitation; FTF: face-to-face; RCT: randomized clinical trial; NRCT: nonrandomized clinical trial; RCS: retrospective cohort study; LBP: low back pain; TKA: total knee arthroplasty; THA: total hip arthroplasty. ^a^Level of evidence. (1) Properly designed RCT or systematic review with meta-analysis; (2) well-designed controlled trial without randomization, prospective study, or comparative cohort trial; (3) case-control study or retrospective cohort study; and (4) case-series or cross-sectional study`.

## Data Availability

All the data related to the manuscript are included within the article.

## Supplementary Materials

Supplementary Materials [1] PRISMA 2020 checklist (supplementary file 1). [2] Search strategy (supplementary file 2).
