# Supplementary material for: Rehabilitation Professional and Patient Satisfaction with Telerehabilitation of Musculoskeletal Disorders: A Systematic Review
Source: Biomed Res Int. 2022 Aug 2;2022:7366063. doi: 10.1155/2022/7366063 (PMC9363217; doi:10.1155/2022/7366063)
Supplement: Supplementary Materials — [1] PRISMA 2020 checklist (supplementary file 1). [2] Search strategy (supplementary file 2). [file 7366063.f1.zip › Supplementry 2.docx]

**Supplemental data**

**Search terms**

We used a variety of key search terms, as listed in the Medical Subject Headings combined with Boolean operators. Search terms were adapted for use in the different databases. Boolean expressions between keywords created a complex search string. Search strings for the research databases differed because of the differences in the indexing methods used by each database. Variations of this string were used**.** PubMed indexes the following under the heading of telemedicine: telerehabilitation, teleradiology, telepathology, and remote consultation.

The following search terms were applied:

The initial search in PubMed was (telemedicine OR telehealth OR telerehabiliataion) AND “Satisfaction AND (Musculoskeletal Disorders).

**Search:** (((((((((telerehabilitation) OR (Telehealth)) OR (telemedicine)) OR (telecunsultation)) OR (telephysiotherapy)) AND (Satisfaction)) OR (experience)) OR (expectations)) AND (Musculoskeletal disorders)) OR (Musculoskeletal Pain)

- telerehabilitation: "telerehabilitation"[MeSH Terms] OR "telerehabilitation"[All Fields]
- Telehealth: "telehealth's"[All Fields] OR "telemedicine"[MeSH Terms] OR "telemedicine"[All Fields] OR "telehealth"[All Fields]
- telemedicine: "telemedicine"[MeSH Terms] OR "telemedicine"[All Fields] OR "telemedicine's"[All Fields]
- Satisfaction: "personal satisfaction"[MeSH Terms] OR ("personal"[All Fields] AND "satisfaction"[All Fields]) OR "personal satisfaction"[All Fields] OR "satisfaction"[All Fields] OR "satisfactions"[All Fields] OR "satisfaction's"[All Fields]
- experience: "experience"[All Fields] OR "experience's"[All Fields] OR "experiences"[All Fields]
- expectations: "expect"[All Fields] OR "expectable"[All Fields] OR "expectance"[All Fields] OR "expectant"[All Fields] OR "expectative"[All Fields] OR "expected"[All Fields] OR "expecting"[All Fields] OR "expects"[All Fields] OR "motivation"[MeSH Terms] OR "motivation"[All Fields] OR "expectancies"[All Fields] OR "expectancy"[All Fields] OR "expectation"[All Fields] OR "expectations"[All Fields]
- Musculoskeletal disorders: "musculoskeletal diseases"[MeSH Terms] OR ("musculoskeletal"[All Fields] AND "diseases"[All Fields]) OR "musculoskeletal diseases"[All Fields] OR ("musculoskeletal"[All Fields] AND "disorders"[All Fields]) OR "musculoskeletal disorders"[All Fields]
- Musculoskeletal Pain: "musculoskeletal pain"[MeSH Terms] OR ("musculoskeletal"[All Fields] AND "pain"[All Fields]) OR "musculoskeletal pain"[All Fields]
